# Updates and controversies for desmoids in familial adenomatous polyposis

**DOI:** 10.1007/s10689-025-00481-9

**Published:** 2025-06-20

**Authors:** Rami James N. Aoun, Matthew F. Kalady

**Affiliations:** Division of Colorectal Surgery, Department of Surgery, The James Comprehensive Cancer Center, The Ohio State University Wexner Medical Center, 410 W 10th Ave, Doan Hall 737B, Columbus, OH 43210 USA

**Keywords:** Desmoids, Familial adenomatous polyposis, Hereditary colorectal cancer, Polyposis

## Abstract

Desmoids are rare non-cancerous fibrous growths with variable behavior ranging from slow indolent growth or even regression, to locally aggressive and progressive tumors that can cause significant morbidity or mortality. Approximately 10–15% of patients with familial adenomatous polyposis (FAP) develop desmoid disease, most commonly located in the abdomen, on the abdominal wall, or in limbs. The majority of desmoids in FAP occur after abdominal surgery. Management is quite challenging and employing a multidisciplinary team at a specialized center is important for success. New treatment modalities have emerged, including tyrosine kinase inhibitors, γ-secretase inhibitors, and ablation techniques, complementing the existing repertoire of therapies such as NSAIDs, anti-hormonal therapy, chemotherapy, radiotherapy, and surgical interventions. Surgery remains the treatment of choice for easily resectable abdominal wall desmoids and intra-abdominal desmoids that cause intractable symptoms, or progressive disease despite alternate therapies, or complications from the invasion of nearby organs. When considering prophylactic colectomies in FAP patients, it’s essential to account for the desmoidogenic potential of surgical interventions, especially in high-risk individuals with a positive family history of desmoids, presence of extracolonic manifestations and carriers of certain genotypes. Given the rarity of the disease and the variability in both anatomical presentation and clinical course, desmoids should be managed by a multidisciplinary team capable of coordinating patient specific care and optimizing treatment options.

## Introduction

Desmoid tumors (DT) are rare mesenchymal derived clonal fibroblastic malignancies. They are benign in nature but exert morbidity through mass effect and local invasion of adjacent tissues and structures. Variability in growth, location, and clinical behavior, coupled with high local recurrence rates and protracted natural history, create a management challenge [1, 2]. As a result, establishing standardized treatment guidelines has been difficult, particularly desmoid disease in the setting of familial adenomatous polyposis (FAP). In fact, standards of therapy have been in flux and in constant revision over the last few decades as our understanding of the disease has deepened [–]. Management strategies have evolved from an emphasis on aggressive surgical resection to a more multifaceted approach. This includes non-interventional active surveillance as well as medical therapy (including chemotherapy), ablative procedures, surgery, and radiation [1, 4, 5, 7]. Treatments must carefully balance control of disease burden with the risk of functional impairments, complications, and economic burden [8]. This manuscript will provide a brief overview of desmoid disease in FAP with a focus on some of current controversies in management and offer some practical applications.

## Background

**Fig. 1:**
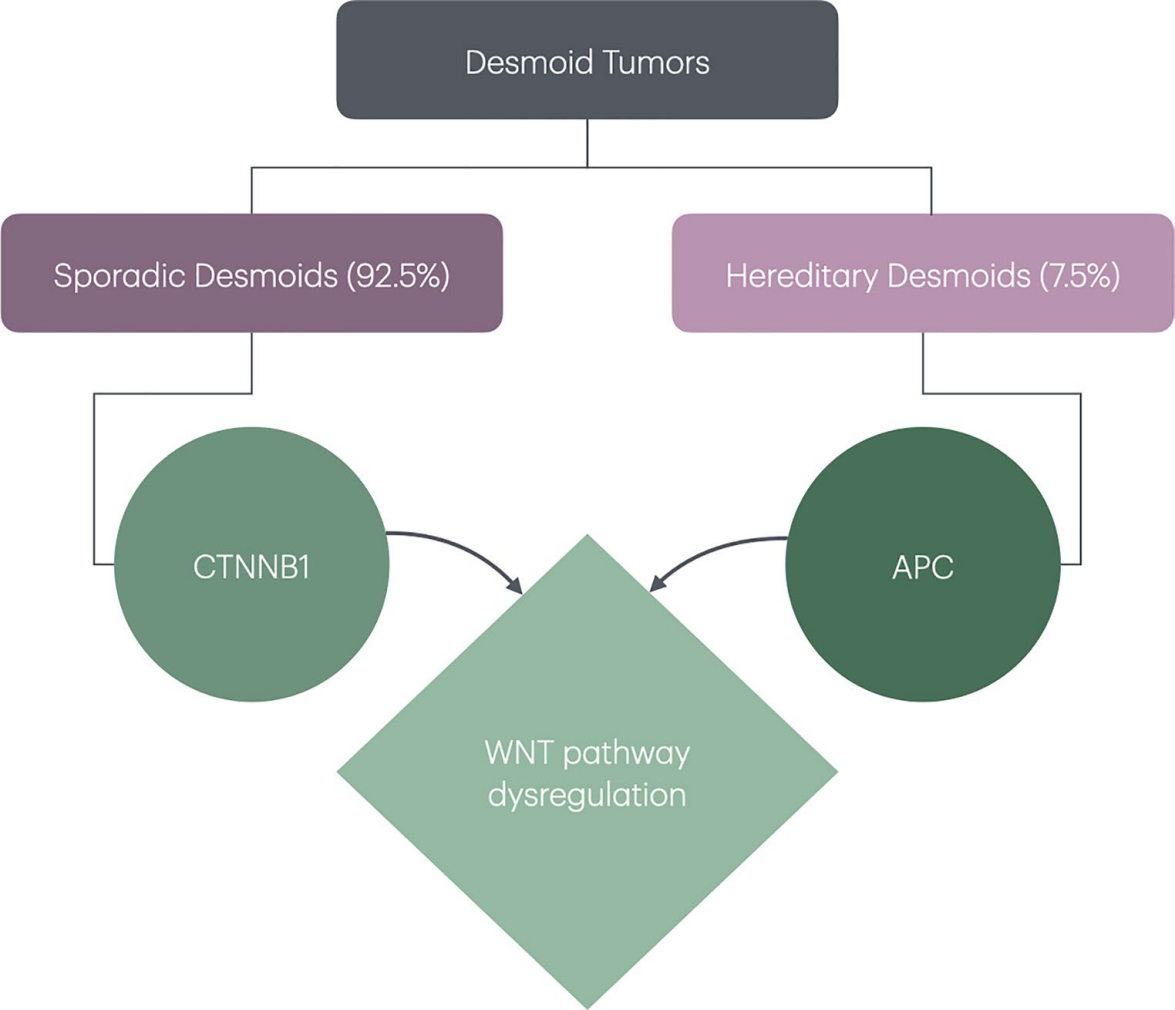
The majority of desmoids are sporadic, and around 7.5% are associated with FAP. Sporadic desmoids are caused by CTNNB1 pathogenic variants and hereditary desmoids are caused by APC pathogenic variants

Desmoid tumors have an average annual incidence of 3.2 per million individuals [9]. The disease has a higher predilection in females with a median age of diagnosis of 38 years, and a range of diagnosis between 3 to 67 years [9–12]. The majority of DTs are sporadic in nature with about 7.5% of all desmoids occurring within a hereditary syndrome such as FAP [13–15]. (Fig. 1**)** For patients with FAP, about 12% will develop desmoid disease, with some studies reporting a cumulative risk as high as 20.6% [16, 17] The risk of DT in FAP is 852 times higher compared to the general population [18]. Both sporadic and FAP related desmoids are driven by molecular dysregulation of the WNT signaling pathway [19–21]. While sporadic desmoids are typically associated with stabilizing pathogenic variants in the β-catenin gene (*CTNNB1*) [19, 20, 22]desmoids in FAP are generally caused by deactivating pathogenic variants in the *adenomatous polyposis coli* (*APC*) gene, which in turn leads to the disinhibition and accumulation of cellular β-catenin [15, 19]. (Fig. 1**)** In FAP patients, and following Knudson’s ‘two-hit’ hypothesis, loss of the second *APC* gene leads to the development of both colorectal cancer and desmoids [23]. 

Desmoid disease represents significant morbidity and mortality in FAP patients, ranking only behind colorectal cancer and duodenal cancer. These desmoids exhibit a distinct anatomical distribution, with a higher incidence in the abdominal wall and intra-abdominal regions which anatomically account for ~ 50% of all DTs [9, 11, 13, 24]. One of the additional challenges of intraabdominal desmoid disease is the morphologic variability. Although desmoids are commonly thought of as masses, desmoid disease often manifests as diffuse, sheet-like fibrosis across the mesentery or bowel. These lesions may not show up on imaging for diagnosis. Additionally, these desmoid sheets are challenging to surgically remove as they are diffuse, infiltrating, and have poorly defined borders. Extra-abdominal desmoids may also occur in FAP, but at a much lower rate when compared to sporadic desmoids [13, 18]. FAP desmoids are more commonly observed in younger patients, with a higher male proportion, and are generally larger, more numerous, and have a greater recurrence rate compared to sporadic desmoids [13, 25]. Other risk factors for their development include prior trauma, implant site, prior radiation site or pregnancy [26–28]. FAP associated DT are more likely to develop at sites of prior surgical intervention and at higher rates when compared to sporadically occurring desmoid tumors [17, 25]. It is no coincidence that the majority of desmoids in FAP patients occur after abdominal surgery, when presumably a “second hit” may occur due to surgical trauma [13, 24, 29, 30]. 

Clinical presentation of hereditary DTs is varied. Symptoms and complications are driven by anatomic location and directly related to infiltrating mass effect on the surrounding viscera and tissues. Superficial abdominal wall desmoids present as palpable and often tender masses. Intraabdominal DTs usually present as abdominal masses and up to one third are asymptomatic [31]. These intrabdominal tumors present as mesenteric plaques or as growing intrabdominal masses. The plaques may induce mesenteric and surrounding tissue puckering, leading to bowel obstruction and hydronephrosis with subsequent renal failure. The intrabdominal masses on the other hand grow and compress intraabdominal structures leading namely to bowel obstruction, perforation with subsequent fistulae formation, intraabdominal infection and hemorrhage [11, 32–35]. Extra-abdominal desmoids can occur anywhere in the body, but are particularly prevalent around the limb girdles and proximal extremities [9, 11]. Intrathoracic DTs may compress the lung parenchyma or esophagus and cause dyspnea, coughing, and dysphagia [36, 37] Intracranial or spinal DTs are rare and manifest clinically with neurological dysfunction or pain [38, 39]. Head and neck DTs specific symptoms may include Horner’s syndrome, cervical radiculopathies and brachial plexus related neurological deficits [40]. 

Church et al. proposed a clinical staging system for intrabdominal and transabdominal desmoids in the setting of FAP [32, 41]. (Table 1) This system is based on four parameters including symptoms, complications, growth rate, and size [32]. Stage 1 desmoids are asymptomatic, < 10 cm and do not have any growth. Stage 2 desmoids are mildly symptomatic, < 10 cm and have no growth. Stage 3 desmoids are moderately symptomatic, 10–20 cm and are slowly growing. Complications include ureter and bowel obstruction. Stage 4 desmoids are severely symptomatic, > 20 cm and are rapidly growing. Complications include sepsis, bowel perforation and hemorrhage. These stages provide a potential framework to guide treatment options.

**Table 1:**
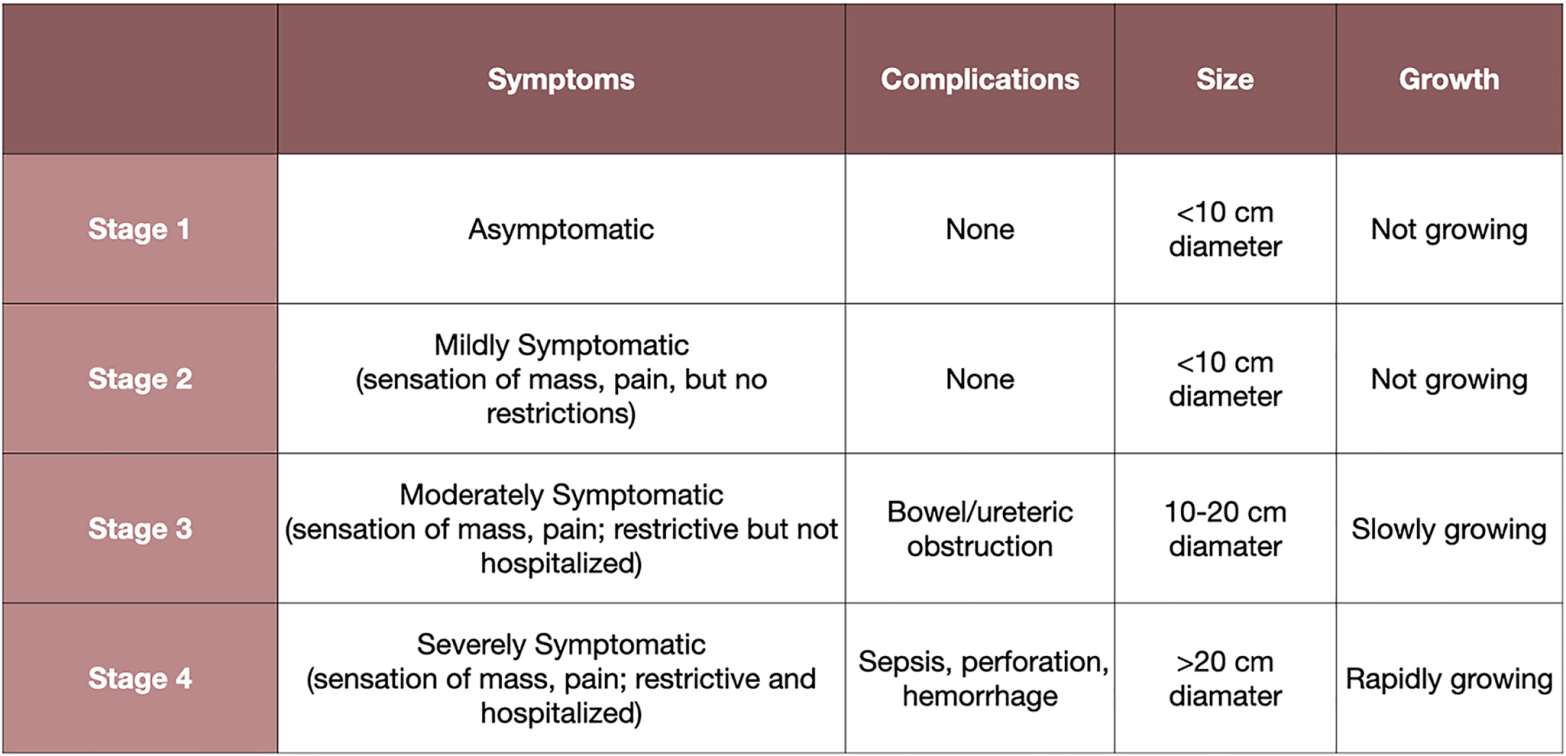
Tidal efficiency along a transect with increasing distance from a tidal channel platform in 1999 and 2019. Uncertainties are standard deviations

### Topics of debate

Due to the rarity and variety of disease, there remains controversy, debate, and variability in the treatments for desmoid disease in FAP. The below discussion addresses some of these topics.

### Role of tumor biopsy for diagnosis

There is debate regarding the need for tissue diagnosis in the management of FAP desmoid disease, depending on the clinical situation. For patients who present with an initial mass suspicious for desmoid tumor without a known FAP diagnosis, biopsy is helpful to confirm diagnosis and allows for somatic tumor testing for variants in *CTNNB1* or *APC*. The Desmoid Tumor Working Group generally recommends biopsy for sporadic DTs [4]. Biopsy is usually performed using a core needle technique with image guidance for intraabdominal masses, or under direct palpation if on a limb or abdominal wall [42, 43]. If biopsy is not feasible, then patients are recommended to undergo germline testing for *APC* pathogenic variant and possibly colonoscopy to evaluate for adenomas. Risks of biopsy include bleeding and infection, although this is rare for limb or abdominal wall mass biopsies. While there is some theoretical concern that biopsy could promote further growth or seeding of desmoids in a population already at risk of developing this tumor, evidence is conflicting [4, 44, 45]. Intraabdominal lesions are often more difficult to access and can be limited by bowel or blood vessels surrounding the lesion. There is a risk of bowel injury in these circumstances. For suspected desmoid tumors in the setting of known FAP, biopsy may not necessarily be needed, particularly for mesenteric masses after colorectal surgery, where these lesions are almost always desmoids. Performing a biopsy in these cases may expose the patient to unnecessary procedural trauma without yielding substantial additional information, especially when an *APC* mutation has already been identified. Recent French guidelines suggest that biopsy of an abdominal or mesenteric mass after colectomy or proctocolectomy is not mandatory and should only be done if there is a strong suspicion of other etiologies [46]. Given the limited added value of biopsy and the potential risks involved, we the authors do not routinely perform a biopsy for abdominal DTs in FAP patients after colectomy.

### Indications and timing of treatment

**Fig. 2:**
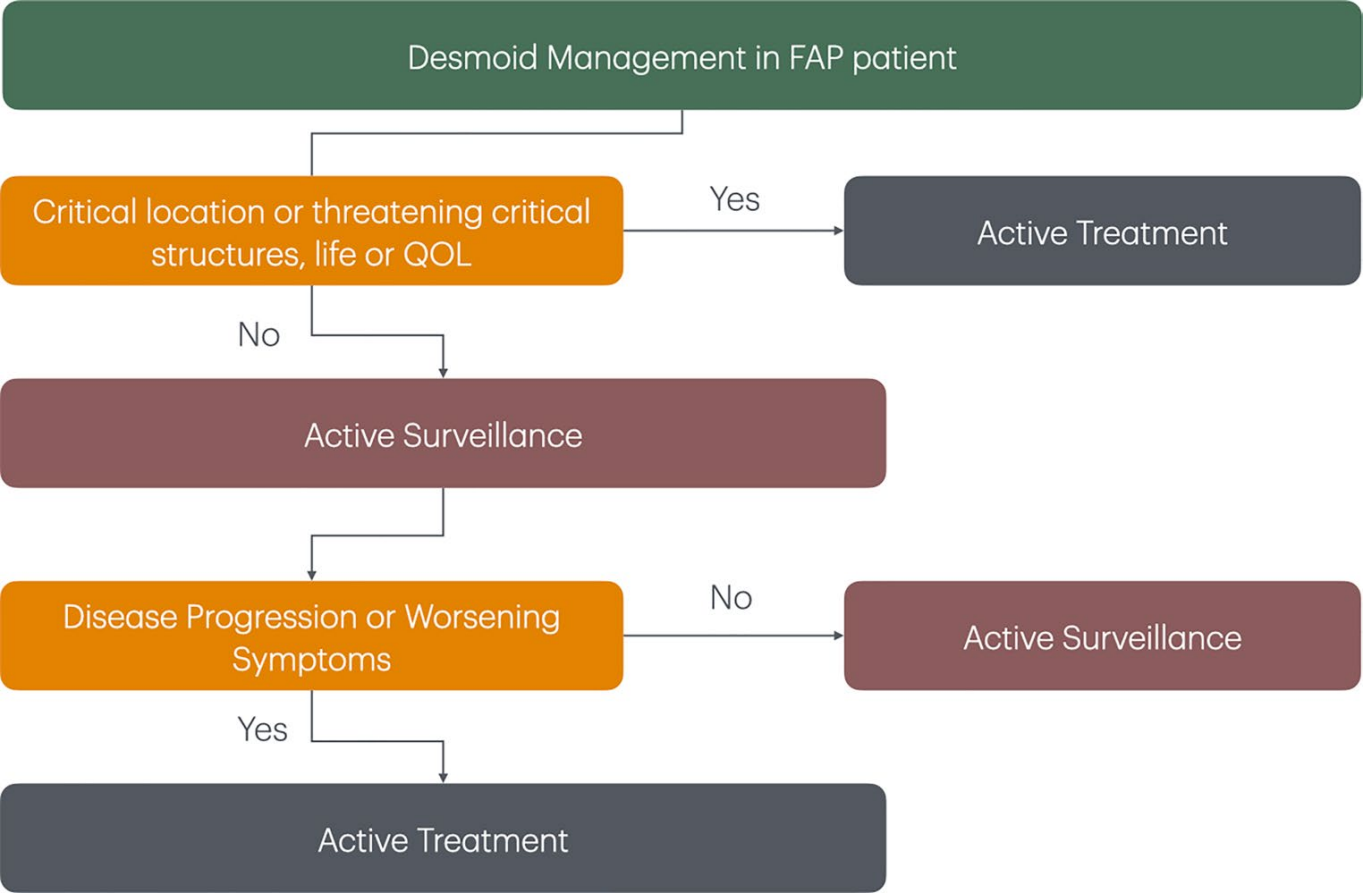
Algorithm of management of desmoids in FAP patients. (QOL = quality of life)

There has been a noticeable shift in the management of DTs in the last few decades [7, 47]. It has been observed that these tumors may often spontaneously regress or experience growth arrest, leading to an indolent disease course [48–50]. Clinical studies have demonstrated that initial observation can yield comparable health outcomes to surgical intervention in cases of DT [49]. Consequently, non-interventional “active surveillance” is now advocated as a first line approach rather than upfront therapy [1, 4, 5, 7, 51]. This is advocated by both the Desmoid Tumor Working Group and the National Comprehensive Cancer Network (NCCN) [1, 4, 5]. Active surveillance does not impact the efficacy of subsequent interventions once indicated [4]. This strategy should include a multidisciplinary evaluation at an expert center with serial assessments and imaging (MRI or CT) performed every 3–6 months after initial diagnostic imaging for 2–3 years extended to every 6–12 months thereafter [1, 4]. A shift from “active surveillance” to “active treatment” should be considered after multiple assessments, typically at least one year after initial diagnosis, with demonstrable progression of disease or worsening symptoms [4]. Active treatment may be indicated at initial diagnosis if the desmoid disease is causing symptoms. This is particularly relevant when the tumor is involving or encroaching bowel, ureter, or head and neck which can significantly impact function, or quality of life. (Fig. 2) [1, 4, 5].

### Types of treatment

Updated guidelines from the NCCN and the Desmoid Tumor Working Group expand the range of therapeutic options available for the “active treatment” phase of DTs [1, 4, 5]. Traditional approaches include medical therapy, surgery, and radiation. Medical therapy ranges from fairly benign treatments such as non-steroidal anti-inflammatory drugs (e.g. sulindac, celecoxib) and anti-hormonal drugs (e.g. tamoxifen, toremifene, raloxifene) to more aggressive chemotherapy including methotrexate, vinorelbine, vinblastine, doxorubicin (including pegylated liposomal formulation), dacarbazine, and hydroxyurea. When evaluating for differential effect of chemotherapeutics comparing mutational status (CTNNB1 vs. APC) no differences were detected. Notably, when comparing responses to chemotherapy based on mutational status (CTNNB1 vs. APC), no significant differences in clinical outcomes were observed [52]. Tyrosine kinase inhibitors (imatinib, sorafenib, pazopanib, nilotinib) have also demonstrated improved success, especially when compared to earlier chemotherapy regimens [53, 54]. Sorafenib is currently the only category 1 tyrosine kinase inhibitor (TKI) recommended by the NCCN for the medical treatment of desmoid tumors [1]. In a phase 3 randomized clinical trial, Gounder et al. reported an 81% 2-year progression-free survival in the sorafenib group, compared to 36% in the placebo group. However, the study did not include a subgroup analysis comparing outcomes between sporadic desmoid tumors and those associated with FAP [53]. The DESMOPAZ phase 2 trial demonstrated improved 6-months progression free survival of another TKI, Pazopanib, compared to combination methotrexate and vinblastine (83.7% vs. 45%).^54^ More recently, new classes of drugs have been approved for desmoid disease. Nirogacestat is a γ-secretase inhibitors and now approved by the FDA for treatment of DTs. The DeFi trial demonstrated a significant improvement in progression-free survival with Nirogacestat compared to placebo, as well as a higher probability of being event-free at the 2-year interval (76% vs. 44%).^54^ Outcomes remained consistent across subgroup analyses, including participants with *APC* somatic mutations (16% in the experimental arm vs. 15% in the placebo arm), *CTNNB1* somatic mutations, and a family history of FAP. However, it is important to council female patients of reproductive age about the potential risks to fertility and the possibility of early menopause [5, 55]. In addition to these established modalities, new and emerging treatments are gaining traction. These include innovative local ablative techniques and vascular therapies like cryoablation, high-intensity focused ultrasonography (HIFU), drug-eluting bead chemoembolization, and trans arterial chemoembolization.

Although most clinicians agree that observation is appropriate for asymptomatic or mildly symptomatic desmoid disease, there is no specific guidance or consensus about initial medical treatment or escalation of therapy. The NCCN and the Desmoid Tumor Working Group give broad guidelines on navigating active therapy choice for DTs [1, 4, 5]. Particularly for FAP associated DTs, both guidelines emphasize that active treatment should be individualized and discussed in a multidisciplinary forum after a period of “active surveillance” or if earlier indications arise. In general, it is rationale to begin with a drug that is effective for the disease burden and has a good safety profile and then escalate to those with more potential side effects.

In recent years, a paradigm shift has emerged in the medical management of desmoid tumors. This is driven by the increasing availability of higher-quality evidence prompting the development of a more defined therapeutic framework. The NCCN and the desmoid working group stop short of recommending a formal medical treatment algorithm, but both bodies emphasize the importance of evidence-based decision-making, with particular attention to the level of evidence and safety profiles of the available drugs [1, 5]. NSAIDs (such as sulindac) and antihormonal therapies, once considered first-line options, are now generally avoided due to limited evidence and reliance on observational data [1, 5]. Instead, nirogacestat and TKIs, particularly sorafenib, are increasingly viewed as preferred first-line therapies. Nirogacestat, in particular, is emerging as a promising first-line agent, supported by impressive clinical outcomes from the previously mentioned Phase 3 DeFi trial and a favorable safety profile [5, 55]. Chemotherapeutics are generally considered second-line therapies. However, they become essential in cases involving symptomatic, rapidly progressing or desmoids with extensive invasion of critical structures that preclude surgical intervention. In such situations, their use is warranted due to the need for a rapid therapeutic response [57]. 

**Fig. 3:**
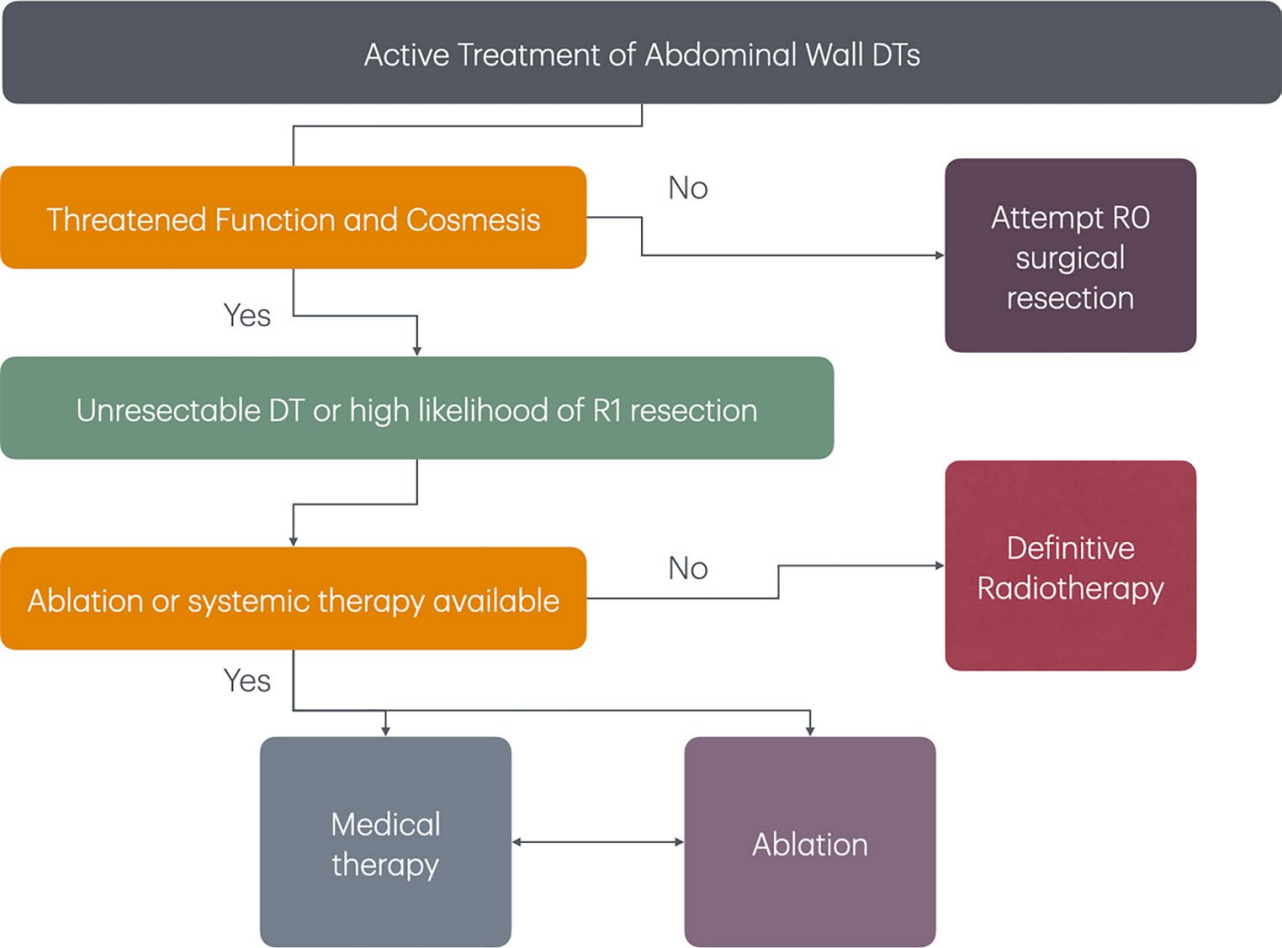
Algorithm of “active treatment” for abdominal wall DTs in FAP patients

**Fig. 4:**
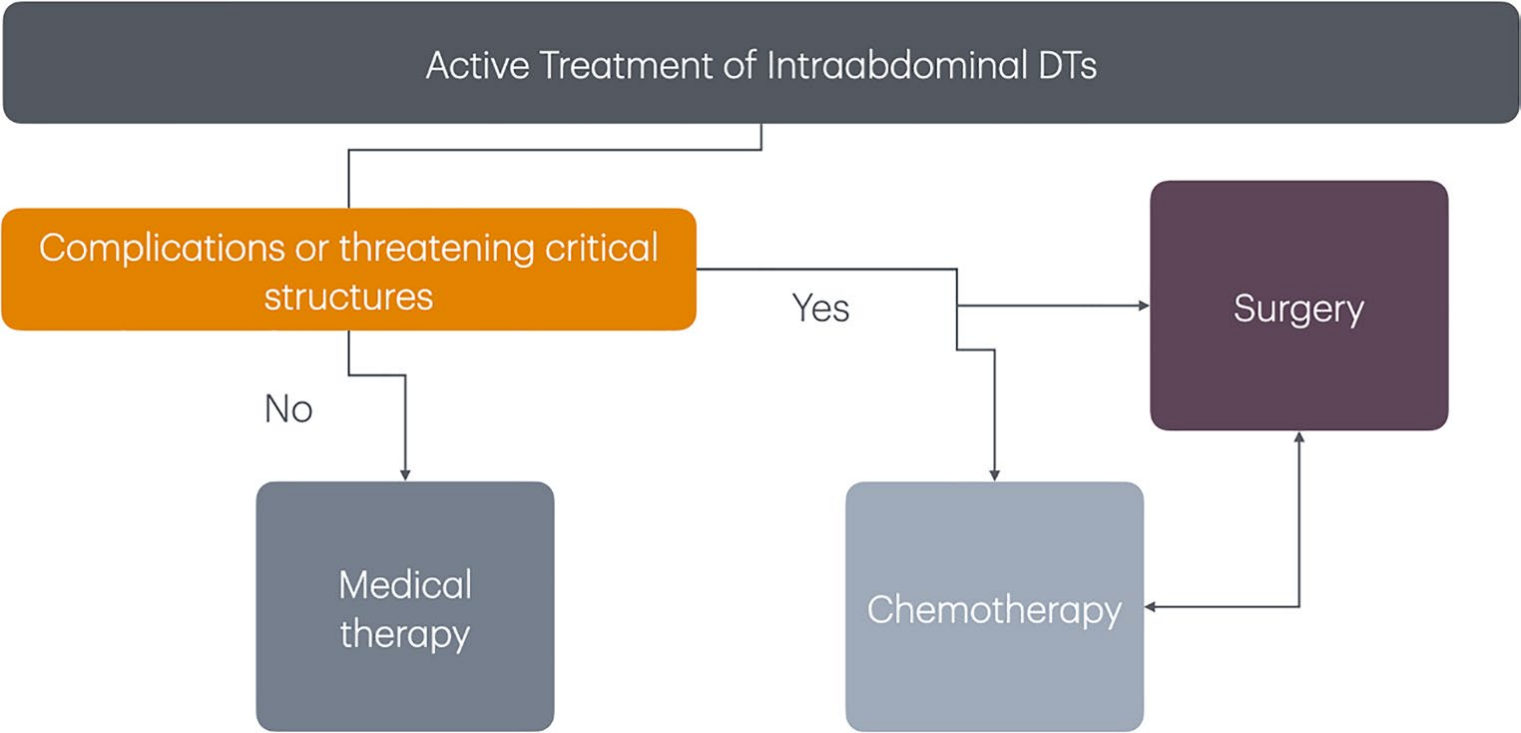
Algorithm of “active treatment” for intraabdominal DTs in FAP patients

Surgery, when able, is an effective treatment in the correct clinical setting. Surgical resection remains the first line treatment for abdominal wall desmoids that can be safely resected [1, 4, 5]. This situation includes high likelihood of an R0 resection without functional or cosmetic compromise. Conversely, if these criteria are not satisfied, then other medical options should be first line treatment. A proposed algorithm is shown in Fig. 3. For intraabdominal desmoids, medical therapy is almost always the first consideration. Surgical resection is often challenging and not able to achieve resection due to involvement of the small bowel mesenteric root. In addition, surgery itself tends to lead to future desmoid formation. Thus, surgery should be reserved for disease that is progressive or cannot be managed with systemic medical therapy. In certain situations, such as bowel obstruction, enterocutaneous fistula, or ureteric obstruction, surgery may be the only palliative option [56]. Surgical intervention for bowel obstruction may include lysis of adhesions, intestinal bypass, or fecal diversion [56]. Surgical options for fistulae may include primary repair, bowel resection but also enteric bypass or fecal diversion. Ureter obstruction may be relieved by ureter stent or even necessitate renal auto transplantation [33]. Radiation is generally not utilized for intraabdominal desmoids due to the toxicity to surroundings structures. A proposed algorithm is shown in Fig. 4.

When extra-abdominal DTs occur in FAP patients, they follow distinct management algorithms [5]. For DTs arising in the extremities, girdles or the chest wall, first line treatment options include medical therapy and ablative techniques. Secondary options involve surgery, radiation and possibly even consideration of isolated limb perfusion. In the case of intrathoracic or neck desmoids, medical therapy remains the first-line treatment, with surgery, radiation, and ablative techniques as secondary options [5]. 

### Impact of desmoids on timing of colorectal surgery

After colorectal cancer, DTs are the second leading cause of death and a major contributor to morbidity in FAP patients [58]. Since the majority develop or are diagnosed after the trauma of surgery, the timing of colectomy or proctocolectomy should be considered within the context of desmoid risk [24, 29]. Delaying colorectal surgery may be beneficial in FAP patients at high-risk of developing DT. These high-risk populations include females, individuals with family history of desmoid disease, presence of extracolonic manifestations, and carriers of specific *APC* gene loci mutations (usually towards the 3’ of codon 1400) [30, 35, 59–61]. First degree relatives of FAP patients with DTs were at much higher risk of developing DTs when compared to second or third degree relatives (25% vs 11% vs 8%).^18^ A desmoid risk prediction model has been proposed based on the above characteristics [35]. Patients with a high-risk score have an 83% chance of developing desmoids while people with a low risk score had only a 5% risk. Regardless of the risk of desmoids, the ongoing challenge is that FAP patients face a 100% risk of developing colorectal cancer without prophylactic surgery [62]. Thus, it is critical to balance prophylactic colorectal cancer (CRC) prevention via colectomy against the desmoidogenic risks brought on by abdominal surgery. Many centers recommend colectomy or proctocolectomy at age 18 regardless of polyp burden, but this practice without consideration of other factors such as quality of life or desmoid risk is not advised. There is no absolute number of polyps or a predetermined age at which time a patient must undergo colectomy. In high-risk patients, it may be prudent to continue with colonoscopic surveillance with aggressive endoscopic polyp control as able. Surgery should only be offered when absolutely indicated, such as in cases of cancer or uncontrolled polyp burden.

### Impact on extent of colorectal surgery on desmoids

Currently, there are no standardized guidelines to guide surgical decision making in patients with FAP who are at risk of developing desmoid tumors. Given that DT is linked to surgical trauma, several studies have aimed to investigate whether factors such as the extent of colectomy and the surgical approach (open vs. minimally invasive) impact desmoid formation. The theoretical premise suggests that a less extensive surgical procedure with minimal tissue manipulation, less tension on the small bowel mesentery after anastomosis may result in a lower incidence of DT following surgery.

The primary options for prophylactic surgical intervention in FAP patients are total abdominal colectomy with ileorectal anastomosis (TAC/IRA), subtotal colectomy and ileosigmoid anastomosis (STC/ISA), and total proctocolectomy (TPC), with restorative ileal pouch-anal anastomosis (IPAA) or end ileostomy (EI). All of these surgeries can be done via laparotomy or using a minimally invasive approach. The effect of the surgical extent and approach remains debated. One of the largest experiences to date, from Sommovilla et al., describes higher desmoid formation after TPC/IPAA compared to TAC/IRA.[63] After a median follow-up of 116 to 120 months, the study found that symptomatic desmoids developed in 46.5% vs. 10.9% of patients who underwent open TPC/IPAA vs. TAC/IRA, and in 35.6% vs. 19.2% of patients who underwent laparoscopic TPC/IPAA vs. TAC/IRA. Vitellaro et al. reported similar findings. Their cohort, which involved 672 FAP patient undergoing prophylactic surgery, also demonstrated that open surgeries are more desmoidogenic compared to laparoscopic approaches [64]. Conversely, a recent meta-analysis by Aelvoet et al., showed similar desmoid formation rates between TPC/IPAA and TAC/IRA.^29^ There was also no statistically significant difference in the postoperative DT incidence when comparing open to laparoscopic approaches or extent of surgery [29]. Upon a more granular examination of the data, this metanalysis included heterogenous studies that are based mostly on smaller cohorts and observational retrospective analysis from disparate institutions. Combining lower-level evidence studies with varying methodologies, inconsistent follow up periods, and conflicting outcomes may have contributed to the overall lack of statistically significant findings derived from this pooled data. Prospective randomized studies may be warranted to delineate the true impact of open vs. minimally invasive and TPC/IPAA vs. TAC/IRA surgeries on desmoids in FAP patients. Given the available data and based on surgical experience, the authors favor minimally invasive approach to open for colorectal surgery in FAP. In addition, for patients at risk of desmoid disease, we will aggressively try to avoid pelvic surgery and IPAA by favoring a TAC/IRA. This includes patients who may have more than the “textbook” 20–30 rectal polyps which is normally an indication for proctocolectomy. In this situation, rectal polypectomy as much as able is done before surgery and then surveilled with additional rectal polyp clearing as able. That being said, a significant risk of rectal cancer outweighs the risk of desmoid disease and if a proctectomy needs to be done for cancer, then that decision takes precedence.

### Role of small bowel intestinal transplant for mesenteric desmoids

DT infiltration into mesentery may compromise mesenteric vasculature and intestinal integrity leading to intestinal failure and parenteral nutrition dependance. Resection of desmoids at the root of the small bowel mesentery is not possible without compromising small bowel viability [65]. Small bowel transplant (and more broadly multivisceral transplant) has emerged as a potential solution. This has been seen as a final effort and sort of salvage procedure, however, some centers now discuss and advocate possible early intervention with enterectomy and small bowel transplant early in the course of disease before the patient has exhausted all other treatment options.

Approximately 9–11% of intestinal transplant recipients at specialized centers are FAP patients with intraabdominal desmoids [65, 66]. Canovai et al. report on their cohort of 15 patients that underwent either isolated intestinal transplant (6 patients), liver intestinal transplant (2 patients) or modified multivisceral transplants (7 patients) secondary to extensive desmoid disease [65]. The majority of patients had Stage 4 desmoid disease and required supplemental parenteral nutrition prior to their transplant. Notably, the five-year patient survival was reported at 82% and there were no desmoid recurrences in their cohort postoperatively. In a separate cohort of 9 patients (6 isolated intestinal, 1 liver-intestinal, 2 multivisceral transplants), Chatziperou et al. observed that the majority of their patients achieved parenteral nutrition independence at a median follow-up of 21 months [66]. Moon et al. even demonstrated the feasibility of ex-vivo desmoid resection followed by autotransplantation of the intestine, providing a novel approach to managing desmoid tumors in the context of intestinal transplantation [67]. The role, time and techniques of small intestinal transplant continues to evolve and remains to be seen.

## Conclusion

Approximately 10–15% of patients with FAP will develop desmoid disease. The presentation and disease course are variable, ranging from asymptomatic to fatal. Trends have shifted to more of an observation first approach for desmoids that are not causing significant harm, with escalation of medical or surgical therapy with progression. Newer systemic therapies are promising to improve outcomes and more research and development is underway. Nuanced patient care and management should be discussed in a multidisciplinary forum in specialized tertiary centers.

## Data Availability

No datasets were generated or analysed during the current study.
